# Functional Neurological Disorder: A Decade of Advances in Neurobiology and Treatment Approaches (2015–2025)

**DOI:** 10.1192/j.eurpsy.2026.11029

**Published:** 2026-07-31

**Authors:** A. Wojas, H. Raai

**Affiliations:** 1 Medicine, (CSOM) City University of New York; 2 Psychiatry and Behavioral Health, SBH Health System, New York, United States

## Abstract

**Introduction:**

FND represents a major source of disability within neuropsychiatry, characterized by neurological symptoms that are inconsistent with recognized disease patterns. FND is theorized to represent subconscious conversion of intolerable emotional distress into neurological symptoms, like motor problems and seizures due to disruptions in brain networks. According to DSM-V, psychological factors no longer need to coincide in time with the onset or worsening of a disorder, though psychological stressors may still be noted as a specifier. There has been progress in elucidating neurobiological underpinnings of FND and in developing multidisciplinary treatment approaches, however, integration of mechanistic findings into clinical care remains a critical challenge.

**Objectives:**

Aims to synthesize recent literature (2015-25) addressing the neurobiological mechanisms implicated in FND and the effectiveness of therapeutic interventions, including physiotherapy-based and psychological treatments in adults.

**Methods:**

A literature review was conducted using PubMed for English-language studies published between January 2015 and June 2025. Eligible articles involved adult FND populations and reported either neurobiological findings or therapeutic outcomes. Randomized trials, cohort studies, and systematic reviews were included if they fit inclusion criteria. Data were synthesized to highlight evidence on pathophysiological mechanisms, intervention effectiveness, and remaining gaps in the literature.

**Results:**

Recent neuroimaging and electrophysiological studies consistently demonstrate altered activity and connectivity within sensorimotor, limbic, and salience networks, suggesting impaired self-agency and aberrant top-down attention control as central mechanisms. Although no single biomarker has been validated, reproducible group-level differences support FND’s status not as a diagnosis of exclusion, but rather as a positive-diagnosis entity. Intervention studies, particularly those involving specialized physiotherapy, cognitive-behavioral therapy, and integrated multidisciplinary programs, report moderate improvements in symptom severity and functioning. However, heterogeneity in outcome measures, limited follow-up durations, and small sample sizes constrain generalizability.

**Conclusions:**

Current evidence supports FND as a disorder of dysfunctional brain network integration rather than malingering or purely psychogenic etiology. Modern brain imaging studies show consistent patterns: parts of the salience and limbic networks light up differently in people with FND, and their connections to motor planning areas are altered, meaning symptoms are produced by measurable changes in brain function. Future studies should prioritize standardized outcome metrics, mechanistic biomarkers of treatment response, and cross-disciplinary clinical collaboration to improve patient outcomes.

**Disclosure of Interest:**

None Declared

